# Agronomic performance and consumer acceptability of improved water yam (*Dioscorea alata* L.) varieties in the Republic of Benin

**DOI:** 10.1016/j.jafr.2024.101292

**Published:** 2024-12

**Authors:** Myriame Dansi, Yêyinou Laura Estelle Loko, Jeannette Gbémissola Fakorede, Paterne A. Agre, Judicaël Laly, Abel Amegan, Honorine Ogou, Patrice Adébola, Hounnankpon Yedomonhan, Alexandre A. Dansi

**Affiliations:** a National High School of Applied Biosciences and Biotechnologies (ENSBBA), National University of Sciences, Technologies, Engineering and Mathematics (UNSTIM), Dassa-Zoumé, BP 14, Benin; b Laboratory of Botany and Plant Ecology (LaBEV), Faculty of Sciences and Techniques, University of Abomey-Calavi, 01 BP 4521, Cotonou, Benin; c International Institute of Tropical Agriculture (IITA), Ibadan, Nigeria

**Keywords:** Agronomic traits, Yields, Culinary evaluation, Multilocal trials, Varietal adoption, Water yam

## Abstract

White yam (*Dioscorea rotundata* L.) is widely cultivated, and is a staple food in the Republic of Benin. However, its production is highly sensitive to soil infertility, leading to low yields over the years. In order to address the challenges of land reduction and climate change, it is crucial to introduce more adapted yam varieties to traditional Beninese agriculture. Water yam (*Dioscorea alata* L.) varieties are viable options, as they need less soil fertility and yield more than *D. rotundata*, but have a poor culinary performance. The aim of this study is to assess the agronomic and culinary performance of 15 improved water yam genotypes developed by the International Institute of Tropical Agriculture (IITA) compared to local cultivars. In 2022 and 2023, multilocal trials (4 sites) were performed in the yam-growing areas, and nine villages were selected for culinary evaluation. Linear mixed-effects models and generalized mixed-effects models showed significant differences (p < 0.05) in location, year of experimentation, and certain evaluated agronomic parameters. The improved varieties had a strong likelihood of producing small tubers that could be used as seeds. The agronomic and culinary performance of local water yam accessions has been superior to that of improved varieties. Due to its numerous medium tubers, TDa_1508044 could be introduced for production of yam chips. TDa_1510080, which showed a stable high yield throughout the trial sites, and TDa_1510119, which gave a great number of marketable large-size tubers, showed the best agronomic performance with a yield of more than 25 t/ha. TDa_1510043, TDa_0000194, and TDa_1515030 improved varieties that performed well in both culinary and agronomic ways could be widely adopted by farmers in the yam-growing areas of Benin.

## Highlights

• *Dioscorea alata* is a potential key for improve yam production and food security in Benin.
• Local water yam accessions presented more good attributes than improved varieties.
• TDa_1510119 that give a great number of marketable large-size tubers.
• Three improved varieties that performed well in both culinary and agronomic fields could be vulgarize among farmers.

## Graphical abstract

**Figure:**
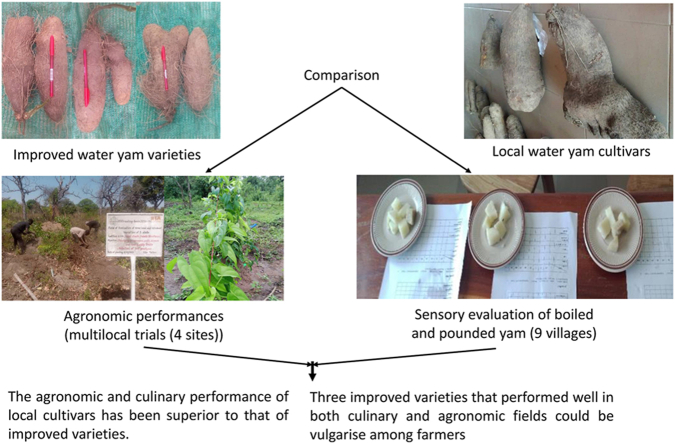

## Abbreviations

TDa: Tropical *Dioscorea alata*
IITA: International Institute of Tropical Agriculture
INRAB: Institut National des Recherches Agricoles du Bénin
ANOVA: Analysis of variance
MCA: Multiple Correspondence Analysis
HSD: Tukey's Honestly Significant Difference (HSD) Test
PCA: Principal Component Analysis

## 1 Introduction

Root and tuber crops are crucial in the sustainable fight against poverty and improving living conditions in rural households in West Africa, specifically in Benin [1]. Yam is a crop of food and cultural importance, and is an important source of income for the Beninese people [2]. Indeed, the Republic of Benin is the fourth largest yam producer in Africa with an estimated per capita consumption of 147.93 kg/person/year in 2021 [3]. Among the eleven species of yam cultivated throughout the world, six species (*D. rotundata* Poir., *D. cayenensis* Poir., *D. alata* L., *D. esculenta* Lour., *D. bulbifera* L., and *D. dumetorum* (Kunth) Pax) underlie yam production in Benin with the white Guinea yam (*D. rotundata*) as the most produced and preferred in Benin [2,4]. However, the production of *D. rotundata* is subject to numerous biotic and abiotic constraints, which contribute to a low yield, and lead to significant losses of varietal diversity of up to 30.82 % [5]. Additionally, the production of *D. rotundata* requires high soil fertility. Its production under continuously cultivated land leads to low yields [6]. Providing yam varieties that have acceptable productivity despite soil infertility and have good culinary characteristics to farmers is of utmost importance.

The water yam (*D. alata*) is the most widely cultivated species throughout the world with less demand on soil fertility, and high yield compared to *D. rotundata* [7,8]. Additionally, water yam has the ability to produce in infertile soils with rapid propagation thanks to the development of bulbils [9]. Water yam tubers, which are richest in vitamins and proteins compared to other yam species, have the particularity of having an attractive shape, and a long post-harvest storage period [10]. Unfortunately, water yam production in Benin is based exclusively on Florido variety [4,11]. This variety was introduced in the central region of Benin between 1970 and 2000 from Puerto Rico by the International Institute of Tropical Agriculture (IITA) and the National Institute of Agricultural Research of Benin (INRAB) [12,13]. Therefore, to ensure food security and empower farmers to combat soil infertility, it is urgent to strengthen the diversity of water yam varieties cultivated in traditional Beninese agriculture. Indeed, growing the proper variety is the effective and costless agronomic practice in crop cultivation. Choosing suitable varieties depends mainly on their ability to absorb and utilize nutrients [[14], [15], [16]] as well as adapting to the stressed environmental conditions [17,18], in addition to the potential to compete the various pests [19,20].

The International Institute of Tropical Agriculture (IITA) that holds an important *ex situ* collection of *D. alata* in their genebank, has developed several improved yam varieties [8,21], that can be introduced into Benin. However, it is known that the agronomic performances of yam varieties such as the tuber size, their early maturity and their ability to produce a good quality boiled and pounded yam are the main criteria for farmer preferences [5,22,23]. To ensure widespread adoption by Beninese farmers, it is crucial that these improved water yam varieties deliver agronomic and culinary performances at least equal to the standard values identified by Loko et al. [2]. Indeed, the Florido variety has not been widely adopted by Beninese farmers due among others to the low quality of its pounded boiled tubers, a popular dish across the country [13,24]. Moreover, it has been demonstrated that the texture of pounded yam obtained from florido and other *D. alata* varieties was not as firm as those obtained with tubers of *D. rotundata* [[25], [26], [27], [28]]. In addition, the pounded yam obtained from the Florido variety presents low values with regard to the attributes of elasticity and smoothness [24]. This study aimed to evaluate the agronomic and culinary performance of several improved water yam varieties from IITA, in order to determine which ones are suitable for introduction and popularization in the Republic of Benin.

## 2 Material and methods

### 2.1 Plant materials

Agronomic evaluation was conducted on fifteen improved water yam accessions obtained from the International Institute of Tropical Agriculture (IITA) yam-breeding program and one local water yam accession collected at Tallou village (Table 1). The improved water yam varieties that presented good agronomic performance (yield ≥40 t/ha) were selected for the sensory evaluation. To compare their sensory attributes, two other local water yam accessions collected at Tallou village were added to the experiment.

**Table 1:** List of tested yam accessions.

N°	Agronomic evaluation		Sensory evaluation	
	*Accession*	*Type*	*Accession*	*Type*
01	Sakata Sossohoun	Local	Sakata Kpeguelehoun	Local
02	TDa_0000194	Improved	Sakata Metchessa	Local
03	TDa_1506142	Improved	Sakata Sossohoun	Local
04	TDa_1508044	Improved	TDa_0000194	Improved
05	TDa_1510010	Improved	TDa_1506142	Improved
06	TDa_1510043	Improved	TDa_1508044	Improved
07	TDa_1510080	Improved	TDa_1510010	Improved
08	TDa_1510119	Improved	TDa_1510043	Improved
09	TDa_1510152	Improved	TDa_1510080	Improved
10	TDa_1511008	Improved	TDa_1510119	Improved
11	TDa_1515030	Improved	TDa_1510152	Improved
12	TDa_1515032	Improved	TDa_1511008	Improved
13	TDa_1520002	Improved	TDa_1515030	Improved
14	TDa_1520008	Improved	TDa_1520002	Improved
15	TDa_1520009	Improved	TDa_1520008	Improved
16	TDa_1520050	Improved	TDa_1520050	Improved

### 2.2 Experimental sites

**Fig. 1:**
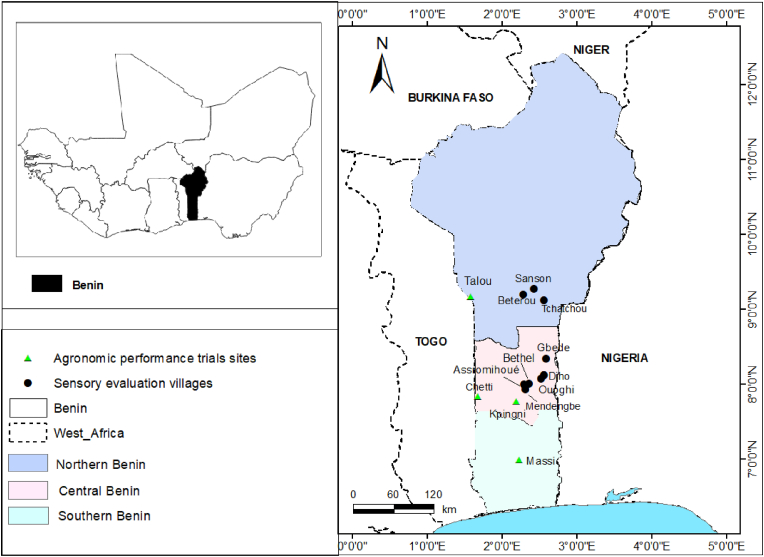
Map of Benin showing trial sites and prospected villages.

The trials were conducted at four sites in the yam-growing area in the Republic of Benin (Fig. 1), and during two cropping seasons (2022 and 2023).

- Massi village (6° 58′ 17″ N, 2° 14′ 27″E), located in the Zou department in the south Benin, and subject to an equatorial climate. The climate is bimodal and varies between dry (from November to March and from mid-July to mid-September) and rainy (from April to mid-July and from mid-September to October) seasons. An average temperature of 26.9 °C and average precipitation of 1048.9 mm are observed. Vertisols and sandy-clayey soils are dominant with a flora dominated by *Diospyros mespiliformis* and *Dialium guineense* [29].
- Dassa (7° 46′ 54″N, 2° 11′ 1″E), and Tchetti (7° 49′ 42″N, 1° 39′ 46″E), are villages in the Collines department in the Centre Benin. In this region, there is a climate of transition between the Guinean climate and the Sudanese climate. It has a dry and rainy season with an average rainfall of about 1200 mm/year. Temperatures range from 21 °C to 36 °C. This area is characterized by ferruginous soils with natural vegetation composed of savannas.
- Tallou (9°7′37″N, 1°40′7.14″E) village in north Benin is characterized by a tropical climate (unimodal) with one dry and rainy season (May to October). The rainfall varies between 1100 and 1200 mm/year and the temperature varies between 27 °C and 35 °C. Air humidity varies by 18 % during Harmattan (December to February) and reaches 99 % in August during the rainy season [30]. (Hydromorphic, ferrallitic soils and lithosols are predominant with vegetation consisting of savannahs and gallery forests.

### 2.3 Experimental design and field management

**Fig. 2:**
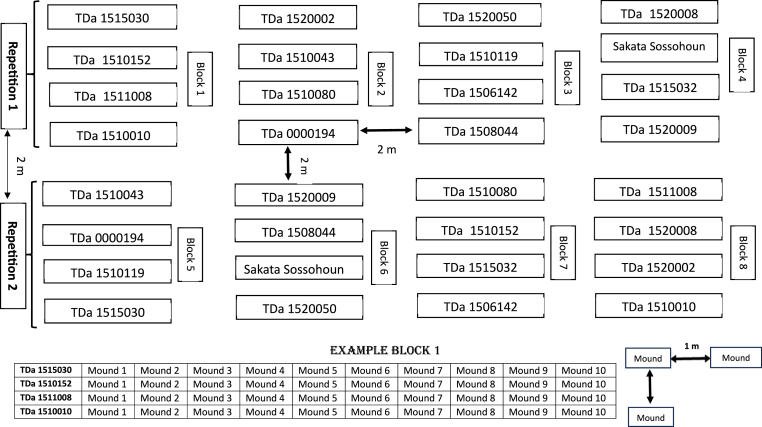
Experimental design used to evaluate agronomic performance of yam accessions.

Experiments were conducted according to the protocol for yam variety performance developed by Asfaw [31]. The experiment was conducted in a lattice design with two repetitions and four blocks by repetition (Fig. 2). During the two years, yam seeds have been sown in March (Massi (10th 2022; 24th 2023), Dassa (14th 2022; 25th 2023), Tchetti (15th 2022; 26th 2023), Tallou (17th 2022; 27th 2023)) and harvested at the end of December. In each experiment, a distance of 2 m was maintained between plots and repetitions. Each block was divided into four rows. Each row contained one accession and ten mounds of each yam genotype, with a spacing of 1.0 m between rows and mounds. Tuber setts were sown directly in the field on mounds (60–80 cm high) at a spacing of 1 m × 1 m. No fertilizer or chemical pesticides were applied. Weed control (3) was manually, and the staking was done for all the mounds.

### 2.4 Measurements

At physiological maturity, five yam mounds per genotype were randomly selected by row, and harvested manually after nine (9) months. The collected tubers per plant were counted, and measured (length and width) according to Asfaw [31]. Knowing that, yam tuber size is the main famers’ preference criteria, which determine the type of use (pounded, boiled, dried chips, wassa-wassa … [2]), harvested tubers were classified into three class in function of their size according to Ref. [32]: small (< 15 cm), medium (>15 < 25 cm), and large (>25 cm) (Table 2). We measured the weight of each tuber category per plant and per plot. The calculation of tuber yield was done using 80 % moisture content.

**Table 2:** Description of the agronomical parameters measured.

N°	Characters	Description	Measure's unit
01	Small tubers per plot counting number	Number of small tubers counted per plot at harvest	–
02	Weight of small tubers per plot	Weight of all small tubers harvested per plot	kg
03	Small size tuber length	Average size of small size tubers harvested per plot	cm
04	Medium tubers per plot counting number	Number of medium tubers counted per plot at harvest	–
05	Weight medium tubers per plot	Weight of all medium tubers harvested per plot	kg
06	Medium size tuber length	Average size of medium size tubers harvested per plot	cm
07	Big tubers per plot counting number	Number of big tubers counted per plot at harvest	–
08	Weight of big tubers per plot	Weight of all big tubers harvested per plot	kg
09	Big tuber length	Average size of big size tubers harvested per plot	cm
10	Total tubers per plot computation	Total number of tubers harvested per plot	–
11	Total tubers per plant computation	Total number of tubers harvested per plant	–
12	Total tuber weight per plot	Weight of all tubers harvested per plot	kg
13	Total tuber weight per plant	Weight of all tubers harvested per plant	kg
14	Total tuber yield computation	Yield of all tubers harvested per plant	t/ha
15	Average tuber weight	Average tuber weight	kg

### 2.5 Organoleptic test

For sensory evaluation, 60 untrained consumers were chosen from three villages selected in Savè, Glazoué, and Tchaourou municipalities respectively. In fact, these three municipalities are known as the main production areas of water yam (*D. alata*) and the yam supply basin in Benin [33]. The great majority (52.7 %) of tasters were men adults. In each village, a panel of 20 consumers was constituted based on their willingness to participate. In each village, fresh tubers (10 kg) of each yam accession were hand-peeled, cut into slices (≃ 5 cm of thick), and cooked (100 °C) with 1 L of tap water [2]. Cooking times were recorded because they vary depending on the yam genotypes [34]. Cooked yam samples were pounded during 5 min using a wooden mortar and pestle. Four samples of boiled and pounded yams of each genotype were putted in plastic dishes, and a coded paper was placed in front of each dish. Before the tasting, a short training was done for the tasters to harmonize the manner of scoring or appreciation, filling in the scoring sheet, and especially for the attribution of scores to each sensory character. Each taster received a combination of samples, and invited to score the samples in an evaluation sheet. A glass of water has been used to rinse the mouth between two sessions. Nine pounded yam sensory characteristics (appearance, colour, moudability, strechability, mealiness, texture, aroma, elasticity, and taste), and six boiled yam features (appearance, colour, aroma, taste, stickiness, and texture) were evaluated using a 5-level hedonic scale [2].

### 2.6 Data analysis

Data of tuber characteristics were analysed using a generalized linear mixed effects model with the Poisson family, and those that were continuous were analysed using a linear mixed effects model with random effects. The yam accession with sixteen modalities and the trial site with four modalities (Dassa, Massi, Tallou, Tchetti) were considered fixed, while the block was considered random. The *lmer* and *glmer* functions of the *lme4* package allowed implementing these models [35]. The analysis of variance (ANOVA) was performed to test the global significance of each factor of the agricultural trial [36]. The number of groups for yam accession was determined using the partition with the greatest inertia [37] after performing an ascending hierarchical classification. The adjusted means of the different models were obtained using the lsmeans function of the lsmeans package [38], and presented graphically using the ggplot2 function [39]. The comparison of the measured characteristics on which the factor yam accessions had a significant effect was done with the multiple comparison Tukey test using the *HSD. test* function and pairwise Tukey test using the *TukeyHSD* function of the agricolae package [40]. All analyzes and graphs were performed in the R 4.2.1 software environment [41].

Data of sensory attributes were submitted to the Generalized Linear Ordinal Regression Models to assess the effect of the factor yam genotype on the different features for boiled and pounded yam. The comparison of the average scores of sensory attributes over yam genotype was done using the Tukey HSD test. The barplot was done using *ggplot* function of package *ggplot2* [39] when *MCA* and *fviz_mca_var* functions respectively from packages *FactoMineR* [42] and *factoextra* [43]. The *clm* function of the *ordinal* package [35] allowed to implement these models when the function *HSD. test* of the agricolae package [40] was used for Tukey HSD test. To assess the relationships between the sensory attributes and yam genotype, *averagetable* and *PCA* functions from package *SensoMineR* [44] were used to respectively built average table of sensory attributes score over variety and perform Principal Component Analysis (PCA) for boiled and pounded yams. Finally, factorial analysis on mixed data allowed us to assess the relationship between sensory attributes, variety, commune and ethnic of panellist using *MFAmix* function of package *PCAmixdata* [45]. All analyses and graphs were performed in the R 4.2.2 software environment [41].

## 3 Results and discussion

### 3.1 Agronomic performance

Analysis using linear mixed-effects models and generalized mixed-effects models revealed significant variations (p < 0.05) in 12 out of 15 measured features across experimental sites and years. In contrast, only three features (number and length of small tubers per plot, and average tubers weight) showed significant variations (p < 0.05) depending on yam accessions. The results showed a high variability in the number of tubers as a function of their size category, length, and weight (Table 3). No evaluated parameters showed any significant difference (p > 0.05) between the local variety Sakata Sossohoun and the improved varieties. The number of small tubers per plot varied from 4.62 ± 0.89 (TDa_1510119) to 10.05 ± 1.84 (TDa_1510080). The average length of these small yam tubers was 9.23 ± 1.33 cm (TDa_1510119) to 12.14 ± 0.41 cm (TDa_1515030), with an average weight of 0.53 ± 0.05 kg (TDa_1515030) to 0.68 ± 0.10 kg (TDa_1515032). The average weight of small tubers per plot (5 mounds) ranged from 3.20 ± 0.68 kg (TDa_1510119) to 6.21 ± 1.28 kg (TDa_1510080). Traditionally, farmers preferentially use small tubers with a size between 250 and 1000 g, as seeds because they give an acceptable yield [46]. Therefore, all the evaluated improved varieties have strong potential to meet the seed availability needs of yam producers. In fact, it has been demonstrated that small yam seed tubers of *D. alata* performed more compared to *D. rotundata* [47].

**Table 3:** Measurement (means) of tubers of different yam accessions according to their sizes per plot (5 mounds).

Yam accessions	Small tubers			Medium tubers			Large tubers			Total number of tubers
	Number	Weight (Kg)	Length (cm)	Number	Weight (Kg)	Length (cm)	Number	Weight (Kg)	Length (cm)	
Sakata Sossohoun	7.87 ± 1.24 ab	5.37 ± 1.17a	11.10 ± 0.86a	5.31 ± 0.97 ab	6.72 ± 1.48a	20.35 ± 0.59a	7.35 ± 1.45a	25.71 ± 6.05b	30.07 ± 1.08a	19.63 ± 3.33a
TDa 1510080	10.05 ± 1.84b	6.21 ± 1.28a	10.71 ± 0.89a	4.01 ± 0.59 ab	4.67 ± 0.86a	19.43 ± 1.54a	6.05 ± 0.83a	17.96 ± 2.78 ab	31.41 ± 0.77a	19.51 ± 2.01a
TDa 0000194	8.06 ± 0.98 ab	4.92 ± 0.93a	12.02 ± 0.52a	4.87 ± 1.26 ab	4.57 ± 1.07a	21.13 ± 0.91a	5.75 ± 0.78a	15.81 ± 2.18 ab	32.19 ± 1.54a	18.56 ± 1.99a
TDa 1520009	7.62 ± 1.62 ab	4.78 ± 1.16a	12.11 ± 0.62a	4.87 ± 0.94 ab	8.29 ± 2.18a	20.88 ± 1.67a	4.92 ± 0.71a	14.67 ± 1.60 ab	30.70 ± 0.67a	16.81 ± 2.28a
TDa 1506142	7.56 ± 0.98 ab	5.09 ± 0.98a	11.46 ± 0.42a	4.91 ± 1.02 ab	4.71 ± 1.13a	21.50 ± 1.29a	5.50 ± 0.65a	15.70 ± 1.69 ab	30.04 ± 0.85a	16.75 ± 1.79a
TDa 1510152	7.00 ± 1.00 ab	3.66 ± 0.83a	10.44 ± 1.00a	4.42 ± 1.09 ab	4.12 ± 1.23a	18.96 ± 0.75a	5.37 ± 0.49a	14.75 ± 1.59 ab	28.08 ± 1.13a	16.63 ± 2.14a
TDa 1510010	7.00 ± 1.36 ab	4.83 ± 1.15a	10.89 ± 1.07a	4.58 ± 1.01 ab	5.75 ± 1.56a	21.76 ± 0.92a	5.81 ± 0.59a	15.19 ± 1.69 ab	30.02 ± 0.97a	16.50 ± 2.26a
TDa 1508044	6.25 ± 1.38 ab	4.61 ± 1.22a	10.89 ± 0.94a	6.21 ± 1.03b	7.49 ± 1.56a	21.84 ± 1.17a	5.06 ± 0.92a	14.92 ± 2.52 ab	31.31 ± 1.40a	16.44 ± 2.65a
TDa 1520008	6.42 ± 1.65 ab	4.90 ± 1.36a	11.32 ± 0.55a	5.07 ± 0.89 ab	6.32 ± 1.33a	21.86 ± 0.54a	5.18 ± 0.71a	17.87 ± 1.52 ab	30.31 ± 1.38a	15.94 ± 2.51a
TDa 1510043	7.71 ± 1.28 ab	5.41 ± 1.01a	9.83 ± 0.77a	4.62 ± 1.59 ab	6.05 ± 1.98a	20.06 ± 1.56a	5.28 ± 0.51a	15.90 ± 2.14 ab	30.64 ± 1.01a	15.50 ± 2.25a
TDa 1520050	7.25 ± 1.06 ab	5.14 ± 1.23a	11.58 ± 0.71a	5.25 ± 1.08 ab	6.90 ± 1.87a	21.79 ± 0.69a	4.62 ± 0.79a	13.19 ± 2.21 ab	29.96 ± 0.78a	15.25 ± 2.42a
TDa 1511008	6.75 ± 0.84 ab	4.35 ± 0.87a	11.74 ± 0.78a	3.50 ± 0.99 ab	3.92 ± 0.92a	20.65 ± 1.13a	6.12 ± 0.79a	14.91 ± 2.44 ab	30.25 ± 1.13a	15.25 ± 1.61a
TDa 1520002	5.88 ± 1.05 ab	4.45 ± 0.92a	10.47 ± 1.19a	4.57 ± 0.94 ab	4.81 ± 1.17a	22.03 ± 0.96a	5.87 ± 0.51a	18.07 ± 2.10 ab	30.98 ± 1.20a	14.55 ± 1.70a
TDa 1515030	7.87 ± 1.41 ab	4.32 ± 0.93a	12.14 ± 0.41a	2.28 ± 0.48a	2.70 ± 0.61a	18.33 ± 2.06a	4.18 ± 0.46a	17.20 ± 2.14 ab	30.02 ± 2.67a	14.18 ± 1.74a
TDa 1510119	4.62 ± 0.89a	3.20 ± 0.68a	9.23 ± 1.33a	3.35 ± 0.87 ab	4.36 ± 1.38a	19.80 ± 1.68a	6.18 ± 0.59a	19.91 ± 1.84 ab	34.13 ± 1.91a	13.75 ± 1.61a
TDa 1515032	7.12 ± 1.75 ab	5.26 ± 1.36a	11.46 ± 0.88a	3.64 ± 0.93 ab	4.29 ± 1.32a	20.30 ± 1.49a	4.91 ± 0.73a	12.58 ± 2.51a	30.33 ± 1.47a	13.63 ± 2.50a

The number of yam tubers classified as having a medium size varied significantly between 2.28 ± 0.48 (TDa_1515030), and 6.21 ± 1.03 (TDa_1508044) depending on the yam genotypes (Table 3). Their length varied between 18.33 ± 2.06 cm (TDa_1515030) to 22.03 ± 0.96 cm (TDa_1520002) with an average weight range of 0.77 ± 0.20 kg (TDa_1506142) to 1.66 ± 0.41 kg (TDa_1520009). This tuber size category is very appreciated by the Beninese populations for the manufacture of yam chips, which are used to prepare a thick paste called *amala* [48,49]. Dried tubers of *D. rotundata*, particularly from the Kokoro varietal group, are traditionally used to make yam chips [12]. However, some studies have shown that the *amala* made with dried Kokoro tubers and that made with Florido (*D. alata*) have similar quality [50,51]. Therefore, the improved variety TDa_1508044, which has presented numerous medium tubers with the second highest average weights per plot (7.49 ± 1.56 kg), could be introduced into the Donga department, which is the main production area for yam chips in Benin [5,22].

In Benin as in the entire in West African region, the first farmers’ preference criterion for the adoption of a variety is the obtaining of large-sized yam tubers serving as offerings in various traditional ceremonies [12,52,53]. The highest number (7.35 ± 1.45) of large-sized yam tubes per plot was obtained with the local variety Sakata Sossohoun. Only the improved variety TDa_1510119 presented tubers with measurements and weights close to those determined by Ref. [2] as a minimum (4.16 ± 0.15 kg per mound, and 36.41 ± 1.22 cm) that must be reached for a yam-improved variety to have the possibility of being adopted by Beninese farmers. However, tubers of all the improved varieties possessed export size (2.0–2.5 kg per tuber, and 15 cm–30 cm), and were marketable [54].

The experimental sites had a significant (p < 0.05) impact on the variability in total tuber yield (Table 4). According to Adjei et al. [55], it is known that there is genotype-by-environment interaction, which lead a differential response of yam cultivars across production environments. The local accession, Sakata Sossohoun, showed great yield variability (CV = 59.86 %) with an average yield of 31.34 ± 6.93 t/ha. The great yield of this local variety suggest that it is important to prospect and evaluate the performance of water yam variety found in the Beninese traditional agriculture to identify potential parent for the national yam-breeding program. The yield of the improved variety TDa_1510080 was stable across the experimental site (with the lowest coefficient of variation of 18.58 %). This could be suggested as the great adaptability of this improved variety according to Ouattara et al. [9]. Furthermore, the TDa_1510080 improved variety achieved a higher minimum yield of 17.93 t/ha, and the highest maximum yield was 42.88 t/ha after the local variety (59.54 t/ha). The highest yield of Sakata Sossohoun (57.78 ± 1.76 t/ha) in Tchetti appears to be excessive for production without mineral fertilizer. However, a similar yield (50 t/ha) was found in Côte d'Ivoire with water yam variety grown in the presence of fertilizer in a forest site [56]. The high yields observed, particularly at Tchetti, are likely due to the site's exceptionally fertile soil, possibly a result of the fallow period implemented there.

**Table 4:** Total tubers yield (t/ha) in each trial site.

Yam accessions	Experimental sites				Average	CV (%)	Minimum	Median	Maximum
	Dassa	Massi	Tallou	Tchetti					
Sakata Sossohoun	47.28 ± 0.22	16.04 ± 6.62	15.42 ± 2.09	57.78 ± 1.76	**31.34 ± 6.93**	59.86	9.42	34.97	59.54
TDa_0000194	33.32 ± 7.83	25.05 ± 2.65	22.22 ± 0.89	30.32 ± 2.82	24.28 ± 2.29	23.82	16.53	26.50	33.15
TDa_1506142	32.04 ± 0.31	18.40 ± 7.10	26.57 ± 0.93	24.63 ± 6.81	23.56 ± 2.44	29.20	11.30	26.57	32.53
TDa_1508044	33.17 ± 3.93	17.86 ± 7.15	15.06 ± 2.80	34.75 ± 1.51	24.48 ± 3.51	41.77	10.71	27.13	36.25
TDa_1510010	35.25 ± 1.76	23.45 ± 2.05	19.40 ± 0.78	26.25 ± 2.28	23.12 ± 2.20	25.00	14.96	24.73	34.90
TDa_1510043	33.37 ± 0.58	18.13 ± 3.33	20.18 ± 0.45	24.58 ± 8.12	22.79 ± 2.67	32.55	14.80	21.04	32.70
TDa_1510080	27.95 ± 1.73	29.32 ± 4.72	23.63 ± 1.09	27.55 ± 6.66	26.57 ± 2.85	18.58	17.93	25.47	42.88
TDa_1510119	34.75 ± 5.69	16.80 ± 4.70	30.13 ± 3.47	28.53 ± 5.76	25.51 ± 2.60	32.15	12.10	27.86	34.30
TDa_1510152	28.08 ± 0.21	9.73 ± 0.93	20.36 ± 3.64	22.26 ± 2.71	17.77 ± 1.96	37.36	8.80	21.77	24.97
TDa_1511008	33.92 ± 1.40	19.20 ± 6.30	23.64 ± 5.60	19.06 ± 1.82	20.81 ± 1.94	33.27	12.90	23.19	31.41
TDa_1515030	40.95 ± 3.84	19.88 ± 1.89	26.50 ± 13.30	24.70 ± 2.84	22.70 ± 1.90	40.53	12.62	24.70	28.79
TDa_1515032	27.28 ± 3.11	17.81 ± 7.68	13.96 ± 6.04	31.07 ± 1.21	20.13 ± 3.85	40.95	7.91	24.83	32.28
TDa_1520002	34.92 ± 2.57	20.96 ± 4.54	20.55 ± 1.80	30.91 ± 1.50	23.21 ± 2.69	27.37	8.08	27.45	32.42
TDa_1520008	39.15 ± 5.11	17.37 ± 8.13	22.00 ± 1.37	18.70 ± 10.70	24.13 ± 3.61	49.77	9.24	24.43	39.28
TDa_1520009	30.65 ± 2.85	15.46 ± 6.31	17.55 ± 5.20	28.70 ± 4.09	24.95 ± 3.61	37.99	9.15	23.68	33.15
TDa_1520050	37.01 ± 0.22	16.22 ± 6.89	12.35 ± 1.60	27.27 ± 5.52	22.08 ± 3.15	49.08	9.33	22.42	35.23

**Fig. 3:**
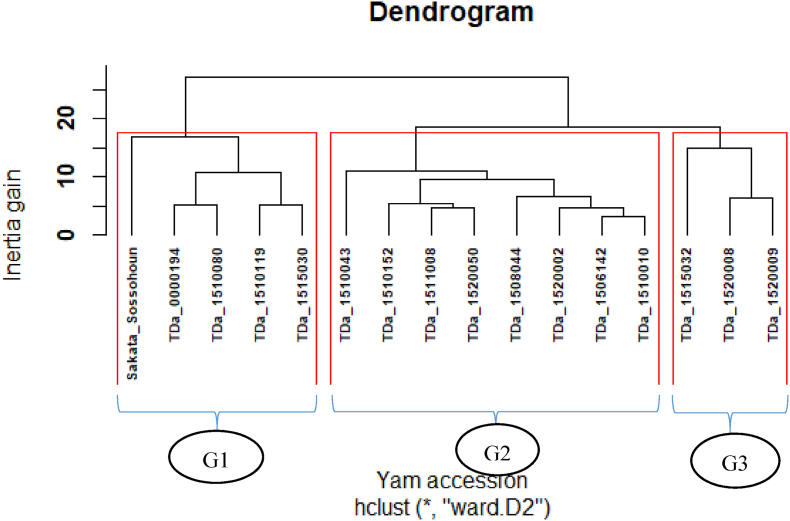
Dendrogram of hierarchical ascending classification of evaluated yam accessions.

Moreover, the hierarchical classification of evaluated yam accessions based on their agronomic performance showed three main groups (Fig. 3). The first group G1 (Sakata Sossohoun, TDa_0000194, TDa_1510080, TDa_1510119 and TDa_1515030) included yam accession producing heavy large-size tubers per plant (Table 5). These accessions’ high market value could significantly increase their potential adoption by Beninese farmers [2].

**Table 5:** Comparison of three group performances.

Variable	G1	G2	G3	P value
Small tubers per plot number	8.68 ± 0.97	7.10 ± 0.32	7.34 ± 0.27	0.165
Weight of small tubers per plot (kg)	6.06 ± 0.59	5.22 ± 0.22	5.26 ± 0.66	0.319
Small size tuber length (cm)	12.22 ± 0.74	10.74 ± 0.55	10.94 ± 0.45	0.241
Medium tubers per plot number	3.95 ± 0.43	3.39 ± 0.32	4.09 ± 0.54	0.430
Weight medium tubers per plot (kg)	5.01 ± 0.53^ab^	4.34 ± 0.38^b^	6.49 ± 0.52^a^	0.038*
Medium size tubers length (cm)	19.10 ± 0.59	15.88 ± 0.91	19.44 ± 3.25	0.119
Big tubers per plot number	6.15 ± 0.34^a^	6.20 ± 0.19^a^	3.97 ± 0.40^b^	<0.0001***
Weight of big tubers per plot (kg)	21.29 ± 1.39^a^	17.74 ± 0.44^b^	13.19 ± 2.06^c^	0.002**
Big tuber length (cm)	30.89 ± 1.15^a^	29.51 ± 0.42^a^	24.34 ± 1.62^b^	0.002**
Total tubers per plot	18.75 ± 1.16^a^	16.71 ± 0.48^ab^	15.22 ± 0.83^b^	0.051
Total tubers per plant	3.81 ± 0.22	3.46 ± 0.09	3.24 ± 0.05	0.074
Total tubers weight per plot (kg)	32.65 ± 1.46^a^	27.44 ± 0.51^b^	24.97 ± 1.40^b^	0.001***
Total tubers weight per plant (kg)	6.53 ± 0.29^a^	5.49 ± 0.15^b^	5.24 ± 0.13^b^	0.003**
Total tubers yield (t/ha)	28.90 ± 1.31^a^	24.36 ± 0.74^b^	23.31 ± 0.53^b^	0.005**
Average tubers weight (kg)	1.92 ± 0.19	1.77 ± 0.08	1.78 ± 0.03	0.635

**Significant level**: * (0.01 < P ≤ 0.05), ** (0.001 < P ≤ 0.01), ***(P ≤ 0.001).

### 3.2 Sensory evaluation

#### 3.2.1 Sensory evaluation of boiled yams

The findings indicated that the boiled tubers from the fifteen yam varieties had a significant difference (P < 00001) in all sensory attributes (Table 6). The improved variety TDa_1510043 was most appreciated by the panellists with an average score of 3.98, compared to TDa_1510119, which showed the lowest average score (1.03 ± 0.02). Previous study carried out by Adinsi et al. [57] showed that boiled tubers of TDa_1510043 improved variety grown in Ubiaja are crumbly, easy to break and meet the requirements of more than 60 % of consumers. However, the local variety Sakata Kpeguelehoun showed boiled tuber yam with high scores for appearance, colour, stickiness, texture, and mealiness features. These sensory features are known as the most important variables contributing to general preferences among consumers [28].

**Table 6:** Sensory evaluation of boiled yam (%) in study area and over variety.

Yam accessions	Overall	Appearance	Colour	Aroma	Taste	Stickiness	Texture	Mealiness
Sakata Kpeguelehoun	3.68 ± 0.08^ab^	2.77 ± 0.06^a^	2.77 ± 0.06^a^	2.52 ± 0.07^ab^	3.03 ± 0.11^ab^	2.68 ± 0.06^a^	2.85 ± 0.05^a^	2.00 ± 0.00^a^
Sakata Metchessa	3.67 ± 0.15^ab^	2.45 ± 0.11^ab^	2.27 ± 0.12^b^	2.07 ± 0.11^cd^	2.4 ± 0.10^c^	2.17 ± 0.12^b^	2.13 ± 0.12^b^	1.67 ± 0.06^b^
Sakata Sossohoun	3.12 ± 0.14^cd^	2.38 ± 0.09^ab^	2.55 ± 0.06^ab^	2.18 ± 0.05^bc^	2.32 ± 0.06^c^	2.10 ± 0.11^b^	2.23 ± 0.12^b^	1.67 ± 0.06^b^
TDa0000194	3.38 ± 0.11^bc^	2.32 ± 0.12^bc^	1.08 ± 0.04^ef^	2.08 ± 0.04^cd^	2.68 ± 0.15^bc^	2.25 ± 0.14^b^	2.93 ± 0.05^a^	1.33 ± 0.06^cc^
TDa1506142	1.70 ± 0.14^efg^	1.45 ± 0.09^ef^	1.00 ± 0.00^f^	1.38 ± 0.08^fg^	1.53 ± 0.11^de^	1.52 ± 0.10^c^	1.70 ± 0.12^c^	1.00 ± 0.00^e^
TDa1508044	1.30 ± 0.06^fgh^	1.00 ± 0.00^g^	1.00 ± 0.00^f^	1.00 ± 0.00^h^	1.00 ± 0.00^f^	1.00 ± 0.00^e^	1.00 ± 0.00^e^	1.00 ± 0.00^e^
TDa1510010	1.63 ± 0.07^efg^	1.52 ± 0.07^e^	1.70 ± 0.07^c^	1.7 ± 0.07^ef^	1.70 ± 0.07^d^	1.00 ± 0.00^e^	1.00 ± 0.00^e^	1.00 ± 0.00^e^
TDa1510043	3.98 ± 0.09^a^	2.70 ± 0.11^ab^	2.88 ± 0.05^a^	2.73 ± 0.09^a^	3.50 ± 0.15^a^	2.45 ± 0.06^ab^	2.70 ± 0.06^a^	**2.00 ± 0.00**^**a**^
TDa1510080	1.82 ± 0.14^ef^	1.52 ± 0.10^e^	1.63 ± 0.12^cd^	1.55 ± 0.11^ef^	1.48 ± 0.10^def^	1.42 ± 0.08^cd^	1.00 ± 0.00^e^	1.00 ± 0.00^e^
TDa1510119	1.03 ± 0.02^h^	1.00 ± 0.00^g^	1.00 ± 0.00^f^	1.00 ± 0.00^h^	1.00 ± 0.00^f^	1.00 ± 0.00^e^	1.08 ± 0.04^de^	1.00 ± 0.00^e^
TDa1510152	1.20 ± 0.06^gh^	1.05 ± 0.03^g^	1.13 ± 0.06^ef^	1.02 ± 0.02^h^	1.18 ± 0.07^ef^	1.07 ± 0.03^de^	2.97 ± 0.02^a^	1.07 ± 0.03^de^
TDa1511008	1.95 ± 0.17^e^	1.60 ± 0.11^de^	1.58 ± 0.11^cd^	1.55 ± 0.11^ef^	1.60 ± 0.11^de^	1.57 ± 0.11^c^	1.78 ± 0.11^c^	1.33 ± 0.06
TDa1515030	2.68 ± 0.06^d^	1.37 ± 0.08^efg^	1.18 ± 0.05^ef^	1.82 ± 0.05^de^	1.43 ± 0.06^def^	1.07 ± 0.03^de^	2.87 ± 0.06^a^	1.67 ± 0.06^b^
TDa1520002	2.02 ± 0.10^e^	1.07 ± 0.03^fg^	1.03 ± 0.02^ef^	1.37 ± 0.06^fg^	1.15 ± 0.05^ef^	1.07 ± 0.03^de^	3.00 ± 0.00^a^	1.00 ± 0.00^e^
TDa1520008	1.45 ± 0.04^fgh^	1.14 ± 0.06^fg^	1.33 ± 0.03^de^	1.13 ± 0.05^gh^	1.27 ± 0.05^ef^	1.24 ± 0.06^cde^	1.35 ± 0.04^d^	1.18 ± 0.06^cd^
TDa1520050	2.82 ± 0.20^d^	1.97 ± 0.10^cd^	2.27 ± 0.12^b^	2.10 ± 0.11^cd^	3.12 ± 0.22^ab^	2.17 ± 0.15^b^	1.02 ± 0.02^e^	1.00 ± 0.00^e^
Pr(>Chisq)	< 0.001***	< 0.001***	< 0.001***	< 0.001***	< 0.001***	< 0.001***	< 0.001***	< 0.001***

Different letters in the same row indicate statistically different averages by the Tukey test (p < 0.05).

**Fig. 4:**
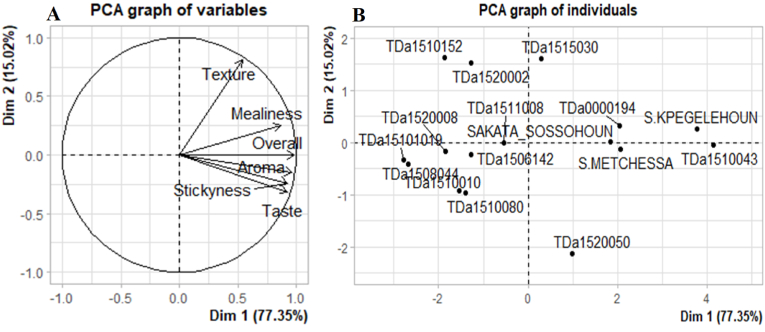
Results of principal component analysis of the variation in sensory attributes of boiled tubers of evaluated yam accessions; A) Circle of correlation of sensory attributes of boiled yams; b) Projection of the studied yam accession in the first factorial plane formed by axes 1 and 2 defined by sensory attributes.

**Fig. 5:**
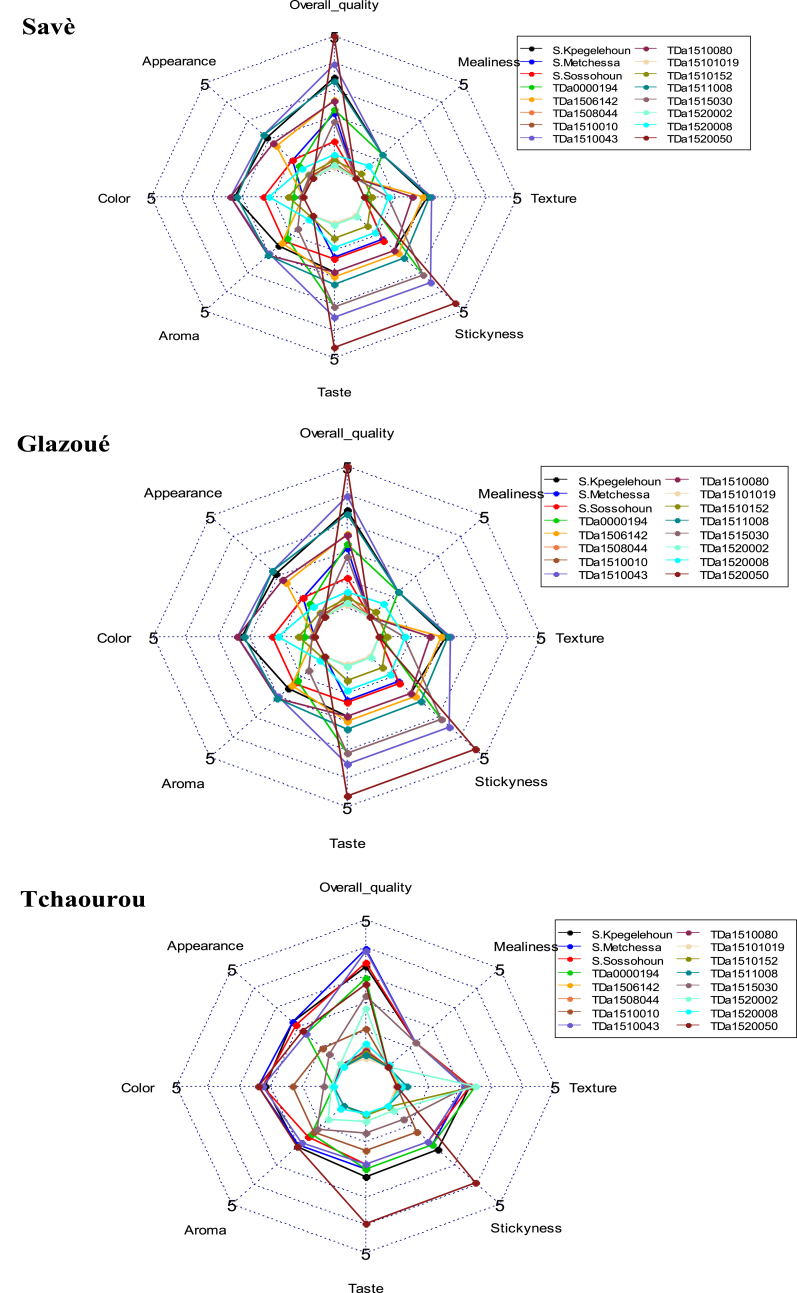
Description of sensory characteristics of boiled yam over the three surveyed districts.

Principal Component Analysis (PCA) revealed that the first two components explained 92.37 % of the information sought. Except for the texture attribute of boiled yams, which was positively correlated with the second axis, all sensory features were positively correlated with the first axis (Fig. 4A). The tasters gave positive feedback on the boiled tubers of all the tested local varieties (Fig. 4B). However, TDa_1510043, TDa_0000194, and TDa_1520050 improved varieties showed the minimum sensorial attributes for boiled tubers defined by Loko et al. [2], thus showing a potential to be adopted by Beninese consumers. Indeed, it appeared that TDa_0000194 accession have sensory qualities generally more appreciated by tasters in Tchaourou. These appreciations were more related to attributes (taste, aroma and stickyness). While, TDa_1520050 and TDa_1510043 were most appreciated by the tasters of Glazoué and Savè municipalities for their taste and aroma (Fig. 5).

#### 3.2.2 Sensory evaluation of pounded yam

Pounded yam from tubers of the local varieties were more appreciated by panellists than those from the improved varieties (Table 7). This is not surprising because it is known that consumers do not like pounded yam from *D. alata* species because of its mediocre quality [58]. However, pounded yam from the improved varieties TDa_0000194, TDa_1510043 and TDa_1515030 were appreciated by panellists respectively for their appearance, aroma, and mealiness.

**Table 7:** Sensory evaluation of pounded yam (%) in study area and over variety.

Yam accessions	Overall	Appearance	Colour	Moudability	Strechability	Mealiness	Texture	Aroma	Elasticity	Taste
Sakata Kpegelehoun	3.13 ± 0.08^c^	3.25 ± 0.06^a^	2.75 ± 0.06^a^	2.13 ± 0.12^b^	1.00 ± 0.00^b^	2.85 ± 0.05^ab^	2.60 ± 0.06^ab^	2.65 ± 0.06^a^	2.72 ± 0.06^ab^	2.80 ± 0.05^a^
Sakata Metchessa	4.05 ± 0.10^a^	3.18 ± 0.05^a^	2.88 ± 0.04^a^	2.77 ± 0.06^a^	1.00 ± 0.00^b^	2.75 ± 0.06^bc^	2.75 ± 0.06^a^	2.60 ± 0.06^a^	2.90 ± 0.07^a^	2.63 ± 0.06^ab^
Sakata Sossohoun	3.55 ± 0.10^b^	3.22 ± 0.05^a^	2.80 ± 0.05^a^	2.10 ± 0.11^bc^	1.00 ± 0.00^b^	2.9 ± 0.06^ab^	2.65± 0.06^a^	2.25 ± 0.06^e^	2.75 ± 0.07^a^	2.45 ± 0.06^b^
TDa0000194	2.92 ± 0.08^cd^	3.25 ± 0.06^a^	1.00 ± 0.00^e^	2.03 ± 0.11^bc^	1.03 ± 0.02^ab^	2.97 ± 0.02^a^	2.40 ± 0.06^d^	2.15 ± 0.05^e^	2.52 ± 0.07^b^	2.57 ± 0.06^b^
TDa1506142	1.00 ± 0.00^h^	1.95 ± 0.03^c^	1.00 ± 0.00^e^	1.00 ± 0.00^d^	1.00 ± 0.00^b^	1.07 ± 0.03^de^	1.18 ± 0.05^d^	1.03 ± 0.02^e^	1.00 ± 0.00^d^	1.00 ± 0.00^e^
TDa1508044	1.25 ± 0.06^gh^	1.40 ± 0.06^f^	1.00 ± 0.00^e^	100 ± 0.00^d^	1.00 ± 0.00^b^	1.00 ± 0.00^e^	1.10 ± 0.04^d^	1.00 ± 0.00^e^	1.00 ± 0.00^d^	1.00 ± 0.00^e^
TDa1510010	1.85 ± 0.05^f^	1.65 ± 0.06^def^	2.05 ± 0.03^c^	1.00 ± 0.00^d^	1.00 ± 0.00^b^	1.00 ± 0.00^e^	1.15 ± 0.05^d^	2.05 ± 0.03^b^	1.00 ± 0.00^d^	2.05 ± 0.03^c^
TDa1510043	2.93 ± 0.09^cd^	2.48 ± 0.07^b^	2.5 ± 0.09^b^	2.18 ± 0.07^b^	1.12 ± 0.04^a^	2.58 ± 0.07^c^	2.25 ± 0.09^c^	2.50 ± 0.07^a^	2.27 ± 0.06^c^	2.45 ± 0.06^b^
TDa1510080	1.20 ± 0.07^gh^	1.90 ± 0.04^cd^	1.00 ± 0.00^e^	1.00 ± 0.00^d^	1.00 ± 0.00^b^	1.00 ± 0.00^e^	1.10 ± 0.04^d^	1.00 ± 0.00^e^	1.00 ± 0.00^d^	1.00 ± 0.00^e^
TDa1510119	1.08 ± 0.04^h^	1.88 ± 0.04^cd^	1.03 ± 0.02^e^	1.08 ± 0.04^d^	1.10 ± 0.04^a^	1.12 ± 0.04^de^	1.13 ± 0.04^d^	1.05 ± 0.03^e^	1.08 ± 0.04^d^	1.03 ± 0.02^e^
TDa1510152	1.20 ± 0.05^gh^	1.90 ± 0.06^cd^	1.00 ± 0.00^e^	1.00 ± 0.00^d^	1.00 ± 0.00^b^	3.00 ± 0.00^a^	1.10 ± 0.04^d^	1.15 ± 0.05^e^	1.00 ± 0.00^d^	1.05 ± 0.03^e^
TDa1511008	1.12 ± 0.04^h^	1.65 ± 0.06^def^	1.02 ± 0.02^e^	1.03 ± 0.02^d^	1.05 ± 0.03^ab^	1.27 ± 0.06^d^	1.08 ± 0.04^d^	1.00 ± 0.00^e^	1.02 ± 0.02^d^	1.00 ± 0.00^e^
TDa1515030	2.65 ± 0.06^de^	1.75 ± 0.06^cde^	1.25 ± 0.06^d^	1.03 ± 0.02^d^	1.00 ± 0.00^b^	2.8 ± 0.08^ab^	1.05 ± 0.03^d^	1.95 ± 0.03^c^	1.05 ± 0.03^d^	1.55 ± 0.06^d^
TDa1520002	2.45 ± 0.06^e^	1.80 ± 0.05^cd^	1.05 ± 0.03^e^	1.07 ± 0.03^d^	1.00 ± 0.00^b^	3.00 ± 0.00^a^	1.20 ± 0.05^d^	1.55 ± 0.06^d^	1.10 ± 0.04^d^	1.20 ± 0.05^e^
TDa1520008	1.44 ± 0.05^g^	1.57 ± 0.05^ef^	1.05 ± 0.02^e^	1.04 ± 0.02^d^	1.06 ± 0.02^ab^	1.14 ± 0.03^de^	1.05 ± 0.02^d^	1.1 ± 0.03^e^	1.03 ± 0.01^d^	1.04 ± 0.02^e^
TDa1520050	2.8 ± 0.09^cd^	3.2 ± 0.05^a^	2.9 ± 0.04^a^	1.83 ± 0.09^c^	1.00 ± 0.00^b^	1.00 ± 0.00^e^	2.35 ± 0.06^c^	2.65 ± 0.06^a^	2.25 ± 0.06^c^	2.45 ± 0.06^b^
Pr(>Chisq)	< 0.001***	< 0.001***	<0.001***	< 0.001***	< 0.001***	< 0.001***	< 0.001***	<0.001***	< 0.001***	< 0.001***

Different letters in the same row indicate statistically different averages by the Tukey test (p < 0.05).

**Fig. 6:**
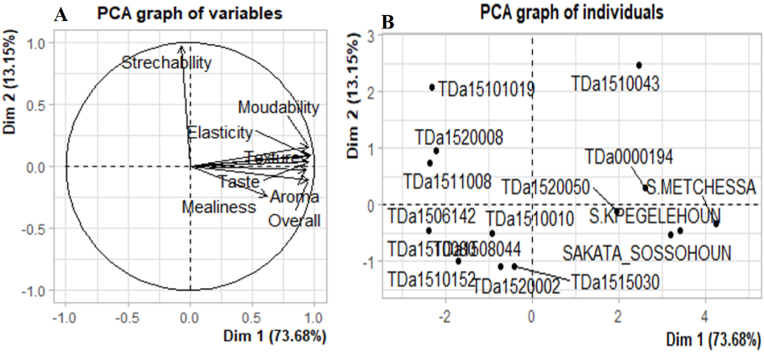
Results of principal component analysis of the variation in sensory attributes of pounded tubers of evaluated yam accessions; A) Circle of correlation of sensory attributes of pounded yams; b) Projection of the studied yam accession in the first factorial plane formed by axes 1 and 2 defined by sensory attributes.

**Fig. 7:**
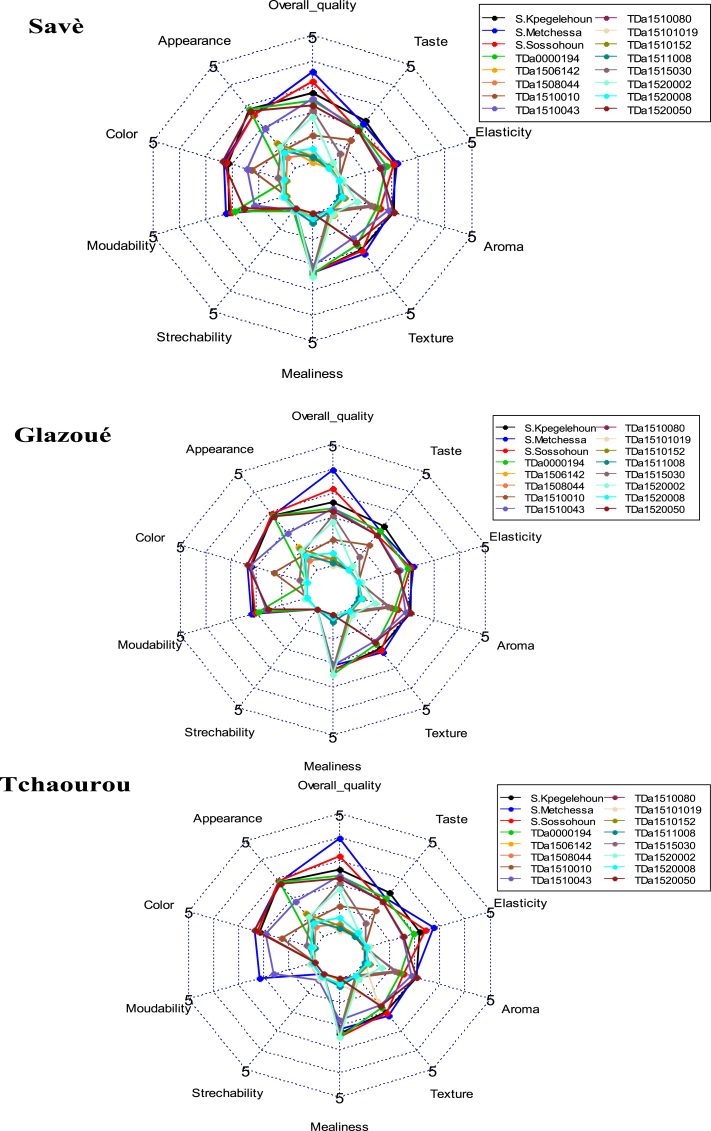
Description of sensory characteristics of pounded yam over the three surveyed districts.

Principal Component Analysis (PCA) performed showed that the first two components explain 86.83 % of the information sought. Except for the strechability of pounded yam, which was positively correlated with the second axis, all sensory features were positively correlated with the first axis (Fig. 6A). The projection of yam accessions in the first two axes (Fig. 6B) shows that several sensory attributes were possessed by all the tested local varieties, followed by the improved varieties TDa_0000194, and TDa_1520050 (Fig. 5B). Indeed, pounded yam of these local water yam accessions were appreciated by consumers (Fig. 7).

## 4 Conclusion

The local varieties of water yam found in traditional Beninese agriculture have shown agronomic and culinary performance better than improved varieties. All the improved varieties showed a strong potential for producing small tubers that could be used as seeds. Due to its numerous medium tubers, TDa_1508044 improved variety could be introduced for yam chips production. However, TDa_1510119 that give a great number of marketable large-size tubers, and TDa_1510080 that showed a stable high yield throughout trial sites have the potential to be adopted by some Beninese farmers. TDa_1510043, TDa_0000194, and TDa_1515030 improved varieties that showed a good culinary and agronomic performance could be vulgarize among farmers throughout yam-growing areas in Benin. Considering locals' varieties performance (best quality of pounded yam and yield), it will be important to identify and popularize others locals’ varieties that have been introduced through exodes and test by farmers in villages near the border for greater and more efficient varietal diversity among farmers.

## Funding

This study was funded by 10.13039/100000865Bill and Melinda Gates Foundation, through RTB project, coordinated in Benin by Prof DANSI Anagonou Alexandre. The funders had no role in study design, data collection and analysis, decision to publish, or preparation of the manuscript.

## CRediT authorship contribution statement

**Myriame Dansi:** Writing – original draft, Visualization, Methodology, Investigation, Formal analysis. **Yêyinou Laura Estelle Loko:** Writing – review & editing, Validation, Supervision, Methodology, Formal analysis. **Jeannette Gbémissola Fakorede:** Visualization, Methodology, Investigation. **Paterne A. Agre:** Validation, Supervision. **Judicaël Laly:** Formal analysis. **Abel Amegan:** Investigation. **Honorine Ogou:** Investigation. **Patrice Adébola:** Project administration, Conceptualization. **Hounnankpon Yedomonhan:** Writing – review & editing. **Alexandre A. Dansi:** Writing – review & editing, Resources, Project administration, Funding acquisition, Conceptualization.

## Declaration of competing interest

The authors declare that they have no known competing financial interests or personal relationships that could have appeared to influence the work reported in this paper.

## Data Availability

Data will be made available on request.
